# Lophotrochozoan neuroanatomy: An analysis of the brain and nervous system of *Lineus viridis*(Nemertea) using different staining techniques

**DOI:** 10.1186/1742-9994-8-17

**Published:** 2011-07-19

**Authors:** Patrick Beckers, Simone Faller, Rudi Loesel

**Affiliations:** 1 Institute of Evolutionary Biology and Ecology, University of Bonn, 53121 Bonn, Germany; 2 Unit of Developmental Biology and Morphology of Animals, Institute for Biology II, RWTH Aachen University, 52070 Aachen, Germany

## Abstract

**Background:**

The now thriving field of neurophylogeny that links the morphology of the nervous system to early evolutionary events relies heavily on detailed descriptions of the neuronal architecture of taxa under scrutiny. While recent accounts on the nervous system of a number of animal clades such as arthropods, annelids, and molluscs are abundant, in depth studies of the neuroanatomy of nemerteans are still wanting. In this study, we used different staining techniques and confocal laser scanning microscopy to reveal the architecture of the nervous system of *Lineus viridis *with high anatomical resolution.

**Results:**

In *L. viridis*, the peripheral nervous system comprises four distinct but interconnected nerve plexus. The central nervous system consists of a pair of medullary cords and a brain. The brain surrounds the proboscis and is subdivided into four voluminous lobes and a ring of commissural tracts. The brain is well developed and contains thousands of neurons. It does not reveal compartmentalized neuropils found in other animal groups with elaborate cerebral ganglia.

**Conclusions:**

The detailed analysis of the nemertean nervous system presented in this study does not support any hypothesis on the phylogenetic position of Nemertea within Lophotrochozoa. Neuroanatomical characters that are described here are either common in other lophotrochozoan taxa or are seemingly restricted to nemerteans. Since detailed descriptions of the nervous system of adults in other nemertean species have not been available so far, this study may serve as a basis for future studies that might add data to the unsettled question of the nemertean ground pattern and the position of this taxon within the phylogenetic tree.

## Background

Nemertea is an undoubtedly monophyletic group of vermiform unsegmented spiralians. Most species are marine, inhabiting a wide range of interstitial, benthic, or pelagic habitats. There are some representatives that have invaded limnic or moist terrestrial environments. To date, about 1280 species have been described [1]. Nemerteans possess a unique structure, the eversible proboscis, to catch and intoxicate their prey organisms. Most benthic nemerteans hunt actively at night at low tide pursuing their prey animals by following them in their tracks [2-4]. For this purpose they use a number of different sensory organs which are mainly situated in the frontal region of the animals [5]. The most conspicuous sensory organs are the cerebral organs. These spherical structures are closely associated with the brain and have been demonstrated to play a role in chemoreception [2,6].

Descriptions of the gross anatomy of the central nervous system of nemerteans were first made in the late 19^th ^and early 20^th ^century. According to these authors, the central nervous system of nemerteans consists basically of a pair of cerebral ganglia and a pair of lateral nerve cords. The cerebral ganglia are arranged as dorsal and ventral lobes which are interconnected by a dorsal and a ventral commissure [7-9]. The cerebral ganglia thus enclose the anterior portion of the rhynchocoel.

Due to morphological characters like the acoelomate body organization, the architecture of the nervous system, the sense organs, and the protonephridial excretory structures, Nemertea were traditionally placed close to Platyhelminthes [10]. In contrast, the fate of the trochoblast cells gives some evidence for including nemerteans into Trochozoa [11]. Moreover, recent molecular studies have produced ambiguous results. Even though none of the molecular based studies found support for a relationship between Nemertea and Platyhelminthes, the placement of Nemertea within Lophotrochozoa varies between different studies [12-17]. Therefore, additional data are necessary to unravel the phylogenetic position of nemerteans.

Searching for novel characters, one promising structure is the nervous system. The methodological backbone of a discipline, that is now being termed "neurophylogeny", has been outlined in a number of publications [e.g. [18,19]. In the last decade neuroanatomical characters have already been used successfully for the inference of phylogenetic relationships within the arthropods [20,21]. Recently, the neuroanatomy of various lophotrochozoan taxa has been studied using immunohistochemical methods [22-28]. Even though immunohistochemical investigations of the larval nervous system of nemerteans have been published [29-31], actually no data are available for adult nemerteans.

In the present study, we revealed the structure of the central and peripheral nervous system of the nemertean *Lineus viridis *using antibodies directed against FMRFamide and serotonin. These two antisera are known to label subsets of neurons in all major animal clades and are frequently used in neuroanatomical studies across the animal kingdom. Therefore, these markers facilitate the comparison of nemerteans to other taxa. Since one aim of this study is to describe the nervous system of a representative of nemerteans in detail, we also used DAPI nuclear labelings and the classical histological Azan staining method to obtain a complete view of the nervous system.

## Results

**Figure 1:**
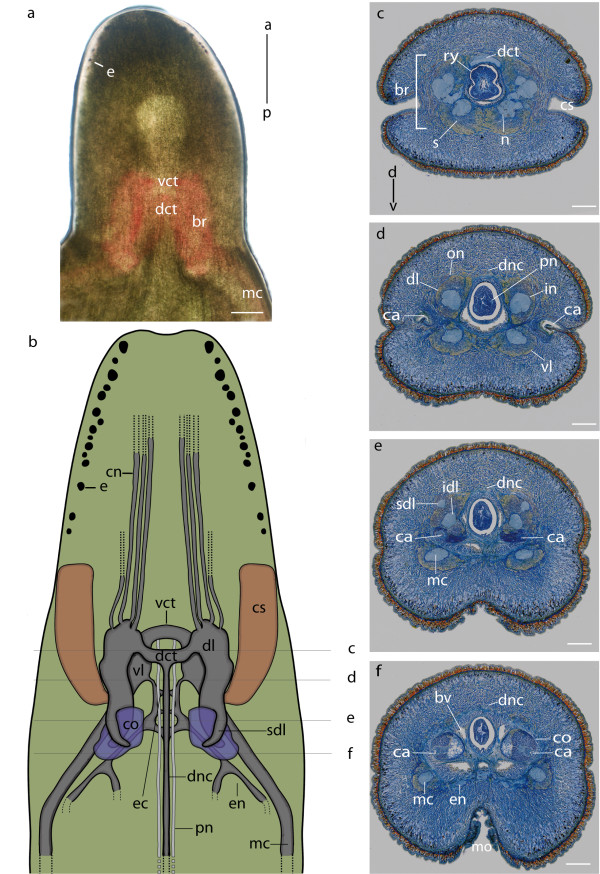
**Overview of the central nervous system of *Lineus viridis***. **a**: Head of a living specimen of *Lineus viridis *(Light microscopy, dorsal view) slightly squeezed, showing the location of the reddish brain (*br*) and the eyes (*e*). **b**: Schematic drawing (dorsal view) of the anterior nervous system of *L. viridis *(the neuronal somata and the cephalic nerves of the ventral part of the brain were omitted). The ventral commissural tract (*vct*) connects the two ventral lobes (*vl*) of the brain, while the dorsal commissural tract (*dct*) connects the two dorsal lobes (*dl*). Posteriorly, the ventral lobes of the brain merge with the medullary cords (*mc*). Medially, the esophageal nerves (*en*) arise from the ventral lobes. A dorsal nerve cord (*dnc*) arises from the dorsal commissural tract. Cephalic nerves (*cn*) extend from the dorsal lobes towards the anterior tip of the animal. The paired proboscidial nerves (*pn*) originate from the ventral commissural tract. **c-f**: Cross sections (Azan staining) of the head of *L. viridis *showing the structures of the central nervous system as shown in **b**. **c**: The brain (*br*) is composed of neuropil (*n*) which is surrounded by neuronal somata (*s*). The brain encloses the rhynchocoel (*ry*). **d**: Paired proboscidal nerves (*pn*) run the full length of the proboscis inside the proboscis musculature. **c-f**: The cephalic slits (*cs*) narrow each into a canal (*ca*) which leads to the cerebral organ (*co*). The cerebral organ (*co*) is attached posteriorly to the inferior branch of the dorsal lobes of the brain (*idl*). *a *anterior; *bv *blood vessel; *d *dorsal; *ec *esophageal commissure; *in *inner neurilemma; *mo *mouth opening; *on *outer neurilemma; *p *posterior; *sdl *superior branch of the dorsal lobe; *v *ventral. Scale bars: a = 500 μm; c-f = 200 μm.

**Figure 2:**
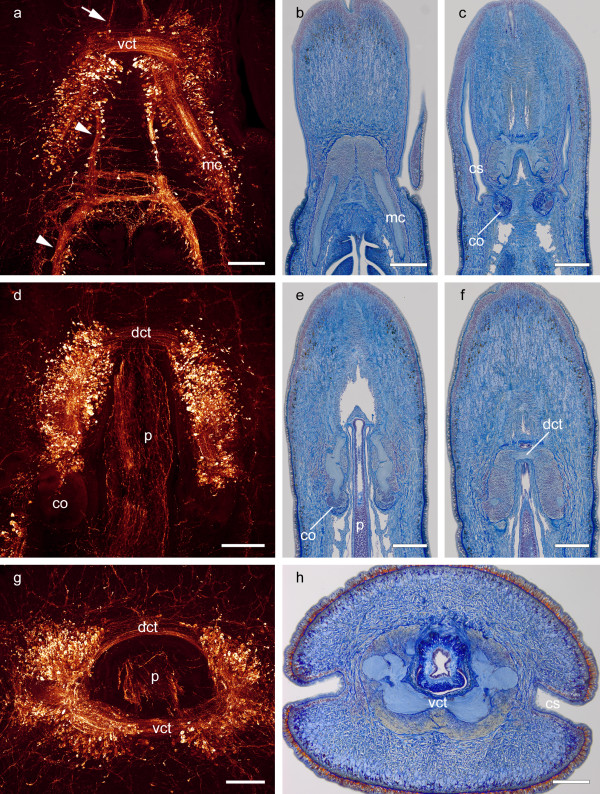
**Comparison of the neuroarchitecture of the brain**. **a-c**: Horizontal sections through the ventral lobes of the brain. The brain is composed of neuropil which is surrounded by densely packed somata. The ventral lobes are connected by the ventral commissural tract (*vct*). Posteriorly, the ventral part of the brain merges with the paired medullary cords (*mc*). Medially, the esophageal nerves (*arrowheads *in **a**) arise from the ventral lobes. Cephalic nerves (*arrow *in **a**) extend from the ventral lobes of the brain towards the anterior tip of the animal. The cerebral organs (*co*) are attached to the brain posteriorly (**c-e**). **d-f**: Horizontal sections through the dorsal lobes of the brain. The dorsal lobes are connected by a dorsal commissural tract (*dct*), as well. The brain is penetrated by the proboscis (*p*) which exhibits a cylindrical plexus in the immunostainings (**d, g**). **g-h**: Cross sections demonstrate that the brain and its commissural tracts form a ring surrounding the proboscis (*p*). *cs *cephalic slits. Scale bars: a,d,g,h = 200 μm; b,c,e,f = 500 μm.

The brain of *Lineus viridis *is located inside the head of the animal. It measures approximately half of the head's width (Figure 1a-d). In the living animal the brain can be identified as a reddish structure in the shape of an inverted U that shines through the semitransparent tissue of the body wall (Figure 1a). It is situated just anterior to the mouth opening. The brain consists of a dorsal and a ventral part. Both parts are clearly separated into two lobes which are connected by a dorsal and a ventral commissural tract (Figure 1a, b). Prominent sensory structures in the head of *L. viridis *are the cerebral organs which are connected posteriorly to the dorsal lobes of the brain and a number of eyes which are arranged along the lateral margin of the animal's head (Figure 1a, b). The ventral lobes of the brain merge with the ventral nerve cords (Figure 1b, 2a, b).

### Brain

**Figure 3:**
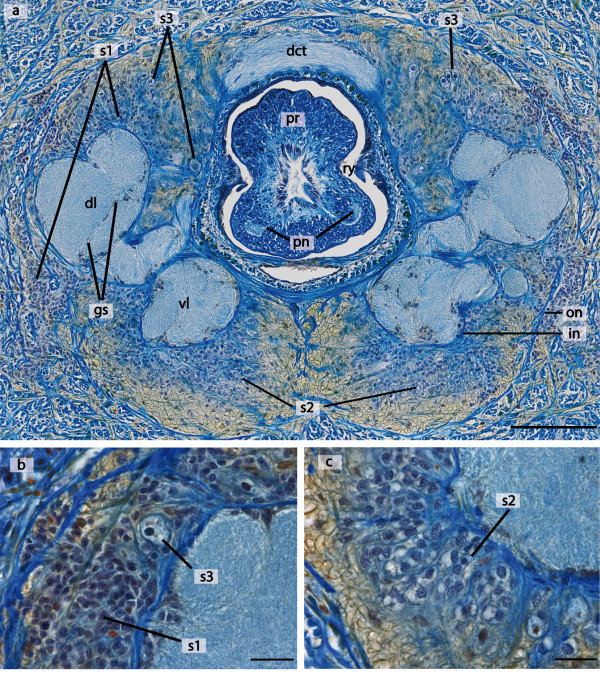
**Cellular architecture of the brain of *Lineus viridis***. **a**: Azan stained cross section of the brain of *L. viridis *showing the location of the neuronal somata surrounding the neuropil of the brain. The neuropil within the ventral lobe (*vl*) and the dorsal lobe (*dl*) is interspersed with glia sheath (*gs*). **b,c**: Higher magnification images demonstrate that the neuronal somata differ in size and can be classified at least in three different types (*s1-s3*). *dct *dorsal commissural tract; *in *inner neurilemma; *on *outer neurilemma; *pn *proboscis nerve; *pr *proboscis; *ry *rhynchocoel. Scale bars: a = 100 μm; b,c = 20 μm.

All parts of the brain are composed of neuropil which is surrounded by a massive layer of densely packed neuronal somata (Figure 1c-d, 2). These somata exhibit variable diameters, falling at least into three size categories (Figure 3). Type 1 somata are clustered and have small nuclei. Type 2 somata are primarily found in the ventral lobe of the brain. They are more slender and elongated than the other two soma types. Type 3 somata are primarily found in the dorsal lobe of the brain, located between the somata of the first type. They differ from the other two soma types because of the size of the nucleus and their pear-like appearance. The cell body layer is separated from the neuropil by an inner neurilemma and is enclosed by an outer neurilemma (Figure 1c-d, 3a). Both neurilemmas entirely consist of extracellular matrix (ECM) clearly indicated by the blue coloration in the Azan staining. While glial sheaths that pervade the cerebral neuropil can be seen in Azan stained sections (Figure 3a), a partition of the brain neuropil was not discernable in the branching pattern of immunolabeled neurons. On each side, the anterior part of each dorsal lobe is connected with the anterior part of the ipsilateral ventral lobe. Both ventral and dorsal lobes are heterolaterally connected by an anterior commissural tract. The more prominent ventral commissural tract is situated anterior to the dorsal one and proceeds below the rhynchocoel, while the dorsal commissural tract connects the two lobes above the rhynchocoel. Accordingly, the frontal part of the brain forms a ring which surrounds the rhynchocoel and the proboscis (Figure 2g, 3a). From here the four individual lobes extend posteriorly and the dorsal lobes bifurcate to form an inferior and superior branch (Figure 1e). An unpaired, dorsal nerve cord arises from the dorsal commissural tract (Figure 1d-f). Cephalic nerves extend from the ventral and dorsal lobes of the brain towards the anterior tip of the animal (Figure 1b, 2a). The paired esophageal nerves emanate posteriorly from the ventral lobes of the brain and are interconnected by the esophageal commissures (Figure 1b, 2a, b). Each esophageal nerve ramifies at the level of the mouth opening. The paired proboscis nerves (Figure 1b, d, 3a) originate from the ventral commissural tract (Figure 1b).

### Cerebral organ

**Figure 4:**
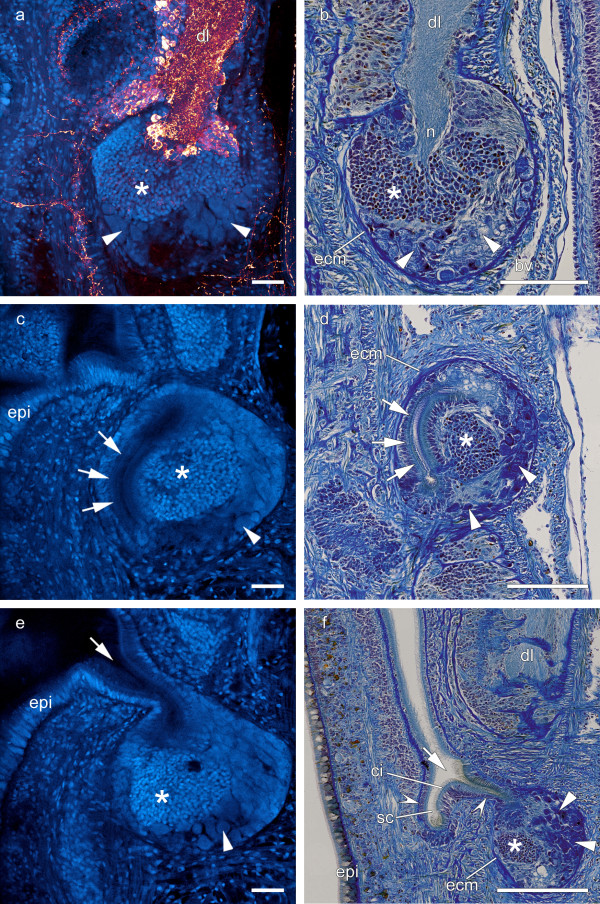
**Morphology of the cerebral organ**. **a**: Overlay image of DAPI nuclear labeling in blue and FMRFamide-like immunostaining in red. **a,b**: The cerebral organ is attached to the dorsal lobe (*dl*) of the brain. Its anterior part is innervated by neurites (*n*) originating from the brain. The remainder of the cerebral organ is filled with densely packed small diameter somata (*asterisk*) and large diameter cells that contain voluminous cell nuclei (*arrowheads*). **c,d**: A canal (*arrows*) runs along the lateral aspect of the cerebral organ. **e,f**: In the most ventral part of the cerebral organ this canal opens into a ciliated epidermal canal (*arrow*) and a sac-like compartment (sc). Here the cells are more slender and densely packed (*open arrowheads*) than in the wider section of the canal. The duct connects the cerebral organ with the environment (**c**). *bv *blood vessel; *ci *cilia; *ecm *extracellular matrix; *epi *epidermis. Scale bars: a,c,e = 40 μm; b,d,f = 100 μm.

Like in all lineids, in *L. viridis *a pair of cerebral organs is posteriorly attached to the dorsal lobes of the brain (Figure 2c-e, 4a, b). While the superior branch of the dorsal lobe rests on the cerebral organ, the inferior branch deeply extends into it (Figure 1b, 4b). Thus each cerebral organ is innervated by immunoreactive neurites originating in the brain. The neurites are surrounded by a cluster of densely packed, small diameter somata (Figure 4a, b). More peripherally, these somata are covered by several layers of cells that are larger in diameter and contain voluminous cell nuclei (Figure 4). Here, the cephalic organ is in close contact to blood vessels, merely separated by extracellular matrix (Figure 1f, 4b). A duct runs along the lateral aspect of the cerebral organ (Figure 4c, d) and opens ventrally into an epidermal ciliated canal (Figure 4e, f). The canal widens and a sac-like compartment branches off (Figure 4f). The lining cells of the narrow canal as well as of the sac-like compartment differ from those being situated in the anterior, wider section of the canal in being more slender and densely packed (Figure 4f). The canal of each cerebral organ widens to a cephalic slit which proceeds longitudinally on each side of the head and connects the cerebral organ with the environment (Figure 1c, d, 2c).

### Nerve cords and peripheral nervous system

**Figure 5:**
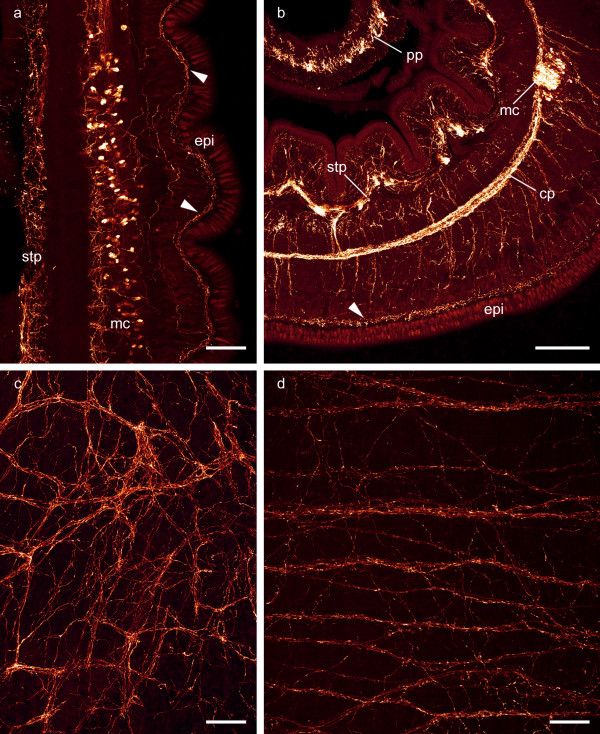
**General anatomy of the peripheral nervous system**. **a**: Horizontal section of the post-cerebral nervous system showing serotonin-like immunoreactive neurites and somata of the medullary cord (mc) as well as the subepidermal plexus (arrowheads) and the stomatogastric plexus (stp). **b**: Transverse section showing FMRFamide-like immunoreactivity in the medullary cord (mc), the subepidermal plexus (arrowhead), the commissural plexus (cp), the stomatogastric plexus (stp), and the plexus of the proboscis wall (pp). **c**: Serotonin-like immunoreactivity in a horizontal section reveals a diffuse arrangement of neurites in the subepidermal plexus (corresponding to arrowheads in **a **and **b**). **d**: FMRFamide-like immunoreactivity in a horizontal section demonstrates that neurites of the commissural plexus are preferentially oriented in circular manner. epi epidermis. Scale bars: a,c,d = 80 μm; b = 200 μm.

The lateral nerve cords originate in the ventral lobes, extend posteriorly, and are embedded between the inner circular muscle layer and the outer longitudinal muscle layer. Serotonin-like immunoreactivity demonstrates that the nerve cord is not only built by immunoreactive neurites. Rather are immunoreactive neurites accompanied by immunoreactive somata which are arranged in a U-shaped manner around the neurites (Figure 1f, 5a, b) and characterize the lateral nerve cord of *L. viridis *as a medullary cord. As in the brain, the neurites are separated from the somata by an inner neurilemma and are enclosed by an outer neurilemma (Figure 1d, 3a). The paired medullary cords are mutually interconnected by circular neurite bundles of the commissural plexus (Figure 5b). The medullary cords as well as the commissural plexus are located between a pronounced subepidermal plexus and a stomatogastric plexus (Figure 5a, b). Numerous interconnections exist between the commissural plexus and both the subepidermal and the stomatogastric plexus. An additional immunoreactive plexus is located in the proboscis wall (Figure 5b). The different plexus are not only characterized by their location within the animal but differ in their arrangement of individual neurites. Neurites of the subepidermal plexus are arranged diffuse and netlike (Figure 5c) while in the commissural plexus the main neurite bundles are predominantly arranged in a highly regular circular fashion (Figure 5d).

## Discussion

In this study, we revealed the neuroanatomy of the nervous system of *Lineus viridis *utilizing antibodies against FMRFamide and serotonin. In addition, Azan stainings and DAPI nuclear labelings were applied to visualize the gross anatomy of the entire nervous system. Applying a combination of different staining methods is a useful approach when trying to describe the neuroanatomy of a species under investigation in as much detail as possible, since each given method reveals only a limited number of aspects of the nervous system. To get an overall impression of the location of all parts of the central nervous system and to understand its spatial dimensions serial sectioning and subsequent Azan staining is inevitable. In comparison, immunohistochemistry marks only a specific subset of neurons in the nervous system and is therefore useful to describe the cellular architecture of the nervous system at a finer resolution.

### Peripheral nervous system

In the peripheral nervous system of *L. viridis *four distinct nerve plexus are present (Figure 5): The subepidermal plexus, the commissural plexus, the stomatogastric plexus, and the proboscidial plexus. The neurites of the subepidermal plexus are arranged in a diffuse net-like manner. In contrast, the neurites of the commissural plexus are arranged in a more regular way. Turbeville and Ruppert [32] described subepidermal and proboscidal nerve plexus in palaeonemertean species. But these authors did not provide any information on the arrangement of the neurites in the plexus. Nevertheless, the available data suggests that a proboscidial and a subepidermal plexus are present in the ground pattern of the nemerteans. Comparing the peripheral nervous system of *L. viridis *with that of non nemerteans it is noticeable that subepidermal plexus have also been reported in platyhelminthes [33,34] and several lophotrochozoan clades such as molluscs [35,36], annelids [22,37], and phoronids [38], albeit they have not been analyzed in detail in these phyla. In ecdysozoans or deuterostomes subepidermal plexus have not been described so far [39].

### Central nervous system

The central nervous system of *L. viridis *consists primarily of the brain and a pair of lateral nerve cords of the medullary type (somata distributed evenly throughout the entire length). Medullary cords are also described for platyhelminthes [34], basal molluscs [26,35,36,40,41], as well as for some polychaetes [37,42]. At current state of knowledge, it is not possible to homologize the medullary cords of these different spiralian taxa.

The brain of *L. viridis *is subdivided into four lobes. Each lobe is composed of a central neuropil which is surrounded by a massive layer of neuronal somata. The neuronal somata are separated from the neuropil by an ECM (inner neurilemma) and the whole brain is also enclosed by ECM (outer neurilemma). The neuronal somata in the brain of *L. viridis *can be classified in three different types (Figure 3). Bürger and Bianchi [7,43] described four different types of somata in the brain of the heteronemertean *Cerebratulus marginatus*. Soma types 1-3 described by Bürger [7] are congruent with our observation in *L. viridis*. In contrast, in palaeonemerteans only one or two types of neuronal somata can be discriminated [[7], own observation of PB]. In some hoplonemertean species the same soma types as described for heteronemertean species are present [7].

This complexity of the brain reflects the active life style of *L. viridis*. The animals are foraging hunters [44] and have an elaborate mating behavior [45] which may explain the well developed nervous and sensory system. Like other lineid species, *L. viridis *shows a higher complexity of the brain than other nemerteans [[5,7], own observation of PB]. This may be related to the characteristics of the cerebral organ of *L. viridis*. Although equally termed sensory organs are also described in hoplonemerteans and tubulanid palaeonemerteans these differ in structure and position. The simplest condition of cerebral organs is found in some palaeonemerteans where they comprise simple lateral sensory pits. In other palaeonemerteans a short duct leads directly to a subepidermal chamber [9]. According to Gibson [9] further development, is achieved by elongation and curving of the duct (cerebral canal). Some palaeo- and the hoplonemerteans have an inner sensory portion of the cerebral canal embedded in a complex of nervous and glandular cells that is connected to the brain [9]. Only in lineid species the cerebral organs correlate with a division of the dorsal lobe of the brain into a blind ending superior branch and an inferior branch that is connected to the cerebral organ. The cerebral organs of *L. viridis *have intimate contact to the environment and are directly innervated by neuritis of the brain (Figure 4a, b). It is very likely that these structures serve as chemo-sensitive organs and allow the animal to orientate in its environment [46]. The structure of the cells in the periphery of the cerebral organ (Figure 4b) suggests that these cells presumably are glandular cells. Together with the proximity of the cerebral organ to the blood vessel this is considered an evidence for its neurosecretory (neuroendocrine) function [6,46-48].

The brain of *L. viridis *is clearly interspersed with glial sheaths. However, these glial partitions which were revealed by the Azan stainings are not reflected by the morphology of immunostained fibers. In this respect, nemertean brain architecture differs from that of other protostome clades with elaborate brains such as vagile annelids and arthropods. Whereas the pattern of immunostaining in the brain of *L. viridis *appears to be evenly distributed with no obvious boundaries, in annelids and arthropods the brain neuropil is clearly divided into several subcompartments [49] which are again termed neuropils. The most prominent of these neuropils in annelids as well as in arthropods are clusters of olfactory glomeruli, the elaborate paired mushroom bodies, and - in arthropods - the unpaired central body whose phylogenetic affinities to the unpaired midline neuropil occasionally described in annelids remains a matter of debate [28,50-52].

Although the brain of *L. viridis *is not subdivided in those different neuropils, there is one structure that resembles the mushroom bodies of annelids and arthropods. The centre of the cerebral organ is formed by a cluster of densely packed small diameter somata which resemble the globuli cells that form the annelid and arthropod mushroom bodies. In the latter two groups the mushroom bodies perceive chemosensory information which is also assumed for the cerebral organs of nemerteans. However, even though the cerebral organs are innervated by immunoreactive neurites, our stainings do not reveal a neuropil that is similar to the typical mushroom body peduncle. The structures of the cerebral organ which implicate a neurosecretory function argue as well against the notion that the cerebral organs might represent modified mushroom bodies. Assuming that the cerebral organ does not represent a modified mushroom body, alternative explanations are conceivable. Another brain associated organ with a comparable location is the nuchal organ of polychaete annelids which shares certain similarities with the nemertean cerebral organ. Nuchal organs presumably play a role in chemosensation, as well. They usually occur pair wise and are innervated directly from the brain [53]. Based on the current morphological data we can neither postulate homology of the cerebral organ to mushroom bodies or to nuchal organs nor exclude the possibility of an independent origin of these structures.

### In search for the urbilaterian brain

Recent molecular fingerprint data indicate that the origin of higher brain centers (e.g. the mushroom bodies) date back to the last common ancestor of all bilaterians and should therefore be present in all bilaterian clades [54]. For example, molecular fingerprint data provide strong evidence for a homology of insect and annelid mushroom bodies [54]. This notion is supported by morphological data which demonstrate that the neuroarchitecture of the mushroom bodies of annelids is in many details identical to that of arthropods [24,52,55]. In addition, the presence of mushroom bodies has been demonstrated for a wide variety of errant polychaetes [28]. In contrast, the complex brain of cephalopod molluscs does not reveal such structures [27]. In our study, we were also not able to clearly demonstrate the presence of higher brain centers like the mushroom bodies in *L. viridis*. Classical studies into the organization of the nervous system of nemerteans [7,8] have never revealed any structure resembling mushroom bodies of annelids or arthropods. Provided that these structures belong to a hypothesized urbilaterian brain, additional immunohistochemical studies in different nemertean species may uncover mushroom bodies or their homologous structures. However, one should consider the possible homology of certain cell clusters in the nemertean brain without having the typical mushroom like structure. In the end, molecular fingerprint studies [54] would be necessary to draw a final conclusion whether centers homologous to mushroom bodies are present outside of Annelida and Arthropoda.

## Methods

Specimens of *Lineus viridis *(Müller, 1774) (Nemertea, Heteronemertea) were collected during field trips in March 2010 on the Isle of Sylt (Germany) during day low tide. Animals were found under stones or shells of *Crassostrea gigas*. To reveal details of the neuroarchitecture of *Lineus viridis*, specimens were analyzed by a combination of immunohistochemistry, DAPI nuclear labeling, and histological Azan stainings. Ten specimens were used for immunohistochemistry and DAPI nuclear labeling and two specimens were used for histological Azan stainings.

### Immunohistochemistry

In principle, immunohistochemistry was performed as described in Heuer and Loesel [24,28]. Animals were anesthetized with a 7% MgCl_2 _solution in seawater. The worms were fixed overnight in 4% paraformaldehyde in 0.1 M phosphate buffer saline (PBS) at 4°C. After fixation all animals were decapitated and the head regions were washed several times in PBS. For vibratome-sectioning (VT1000S, Leica Microsystems, Wetzlar, Germany) the head regions were embedded in a gelatine/albumin medium. After hardening the gelatine/albumin blocks overnight in 14% Formalin in PBS at 4°C, they were cut into sections of 80 μm in thickness. The sections were then washed in PBS with 0.1% Triton X-100 (TX) and pre-incubated overnight in a blocking solution of PBS containing 0.5% TX and 5% normal swine serum (Jackson ImmunoResearch, West Grove, PA). Primary antibodies were added directly to the blocking solution and incubated overnight at room temperature. The primary antibodies anti-FMRFamide (ImmunoStar, Hudson, WI) and anti-serotonin (Sigma-Aldrich, Saint Louis, MO) were both used at a dilution of 1:20000. After incubation with primary antibodies the sections were again washed in PBS with 0.1% TX and were then incubated overnight with the secondary antibody conjugated to fluorophore (Cy3-conjucated goat anti-rabbit; Jackson ImmunoResearch, West Grove, PA) at a dilution of 1:2000 in PBS containing 0.5% TX and 1% normal swine serum. Following removal of the secondary antiserum the sections were incubated with the nuclear marker DAPI (4',6-Diamidino-2-phenylindole, dilactate; Sigma-Aldrich, Steinheim, Germany) at a dilution of 1:1000 in PBS for 10 min. Subsequently, sections were rinsed again in several changes of PBS containing 0.1% TX and then mounted on chrome alum/gelatine-coated glass slides under glass coverslips using Elvanol (mounting medium for fluorescent staining after Rodriguez and Deinhard [56]). Preparations were analyzed with a confocal laser scanning microscope (TCS SP2, Leica Microsystems, Wetzlar, Germany). A helium/neon laser (excitation wavelength 543 nm, detection range 555-700 nm) was used to detect Cy3 fluorescence, DAPI fluorescence was detected with a diode laser (excitation wavelength 405 nm, detection range 410-550 nm). The resulting image stacks were collapsed using the "maximal projection" tool of the TCS SP2 Leica confocal software. These images were finally processed using global imaging enhancement procedures (contrast, brightness) and superposition functions of Adobe Photoshop CS.

### Azan staining

For Azan staining the animals were anesthetized in a 7% MgCl_2_-solution and fixed overnight in Bouin's fixative. The animals were completely dehydrated in an ascending ethanol series (70 - 90%) followed by incubation in Methylbenzoat and Butanol. Afterwards the animals were preincubated in Histoplast (Thermo Scientific, Dreieich, Germany) at 60°C and finally embedded in Paraplast (McCormick Scientific, Richmond, USA). Horizontal and cross sections of 5 μm in thickness were made with a microtome (Autocut 2050, Reichert-Jung, Leica, Wetzlar). Sections were transferred to microscope-slides which were coated with albumen-glycerin to facilitate adherence of the sections to the slides. Sections were stained with Carmalaun and Anilin-blue orange G. After embedding the sections with Malinol (Waldeck, Münster, Germany), slides were analyzed with an Olympus microscope (BX 51, Olympus, Hamburg) which was equipped with a digital camera (CC-12, Olympus, Hamburg) and the dotslide system (2.2, Olympus, Hamburg) which allows an automated scanning of the slices at high resolution. Different neuronal structures can be distinguished in the Azan staining by different coloration: the neurites are dyed grey, the somata are colored in dark purple, and the neurilemma as an extracellular matrix is dyed blue. The schematic drawing (Figure 1b) was derived from a 3D reconstruction (unpublished) using Illustrator and Adobe Photoshop CS4.

The nomenclature used for describing neuroanatomical structures conforms to Richter et al. [57]. According to definitions presented in this glossary, the ganglionated nerve cords are named medullary cords. The dorsal and ventral nerve cords are named dorsal or ventral commissural tracts. The fibrous core of the cerebral ganglion is named neuropil of the brain.

## Competing interests

The authors declare that they have no competing interests.

## Authors' contributions

PB, SF, and RL conceived this study. PB collected the animals and provided the Azan stainings. SF was in charge of the immunohistochemical stainings and of the confocal microscopy. PB, SF, and RL all contributed to writing the manuscript. All authors read and approved the final manuscript.
